# Supplementary material for: Erythropoietin regulates energy metabolism through EPO-EpoR-RUNX1 axis
Source: Nat Commun. 2024 Sep 16;15:8114. doi: 10.1038/s41467-024-52352-z (PMC11405798; doi:10.1038/s41467-024-52352-z)
Supplement: Supplementary file 1 — Supplementary Information [file 41467_2024_52352_MOESM1_ESM.pdf]

Erythropoietin regulates energy metabolism through EPO-EpoR-RUNX1 Axis

Yin W et al.

Supplementary Information

Supplementary Figures

Supplementary Figure 1

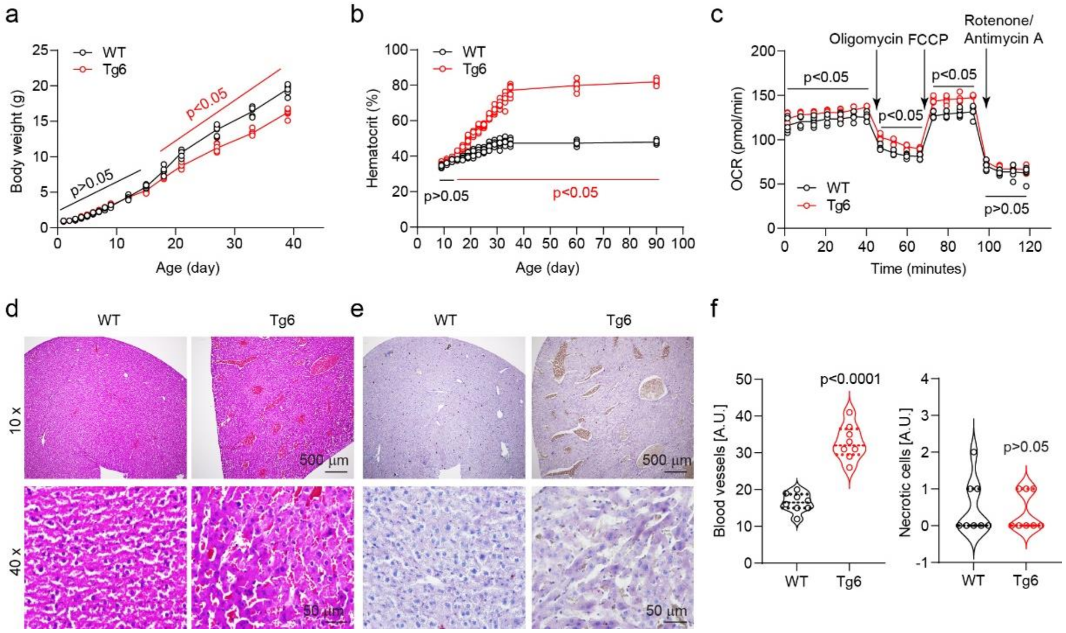

Supplementary Figure 1. Body weight, hematocrit and oxygen consumption of adipose tissue, and liver sections from young male Tg6-mice. a, Body weight of WT control (black) and Tg6 (red) mice from day 0 to 40 days old (n = 7). **b,** Hematocrit (percent) of WT control (black) and Tg6 (red) mice from day 0 to 90 days old (n = 7). **c,** Oxygen consumption rate for adipose tissue from WT control (black) and Tg6 (red) mice. p-values are indicated. **d**, H&E staining of liver sections from control and Tg6-mice reveal intact tissue without signs of necrosis in Tg6-mice. Scale bar: 500 μm (top), 50 μm (bottom). **e**, Oil red O staining (hematoxylin counterstain) of WT-mice and Tg6-mice liver sections shows no fat accumulation. Scale bar: 500 μm (top), 50 μm (bottom). **f**, The notable increased presence of red cells and blood vessels, indicated by quantification of blood vessels (left), without significant increase in necrotic cells (right) are shown for the liver sections from WT-mice and Tg6-mice (n = 8 indicates the number of mice in each group). A.U. means arbitrary units. Scale bar indicated in the figure. Two-tailed unpaired t-test, Two-way ANOVA with Bonferroni's multiple comparisons test. Source data are provided as a Source Data file.

Supplementary Figure 2

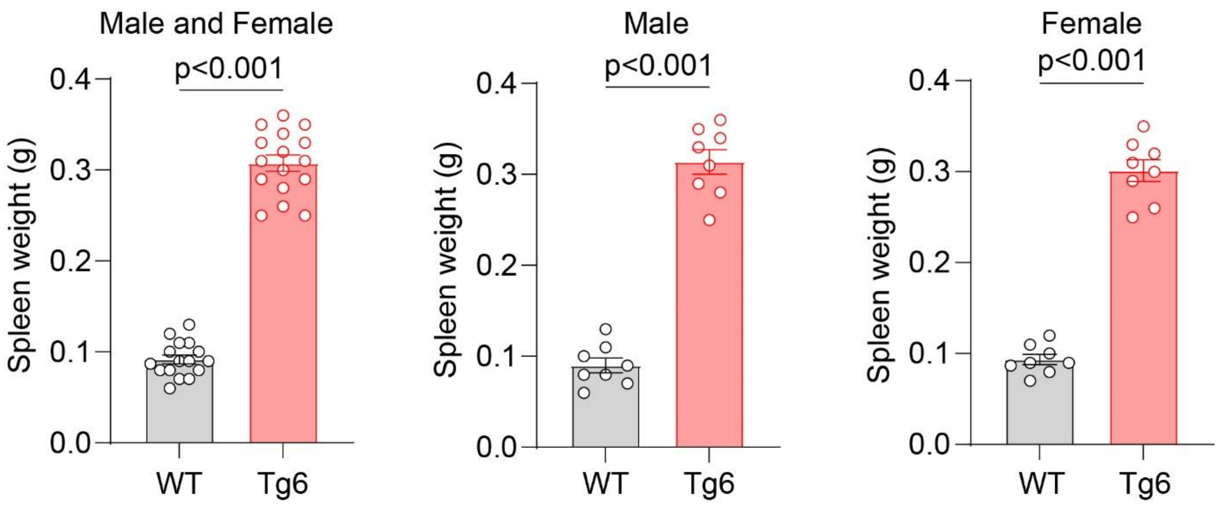

Supplementary Figure 2. Spleen weights of WT and Tg6 mice. Spleen weight was assessed in males and females at 2 months of age for WT and Tg6 mice. (male and female: n = 8 indicates the number of mice in each group). p-value are indicated. Two-tailed unpaired t-test. Source data are provided as a Source Data file.

Supplementary Figure 3

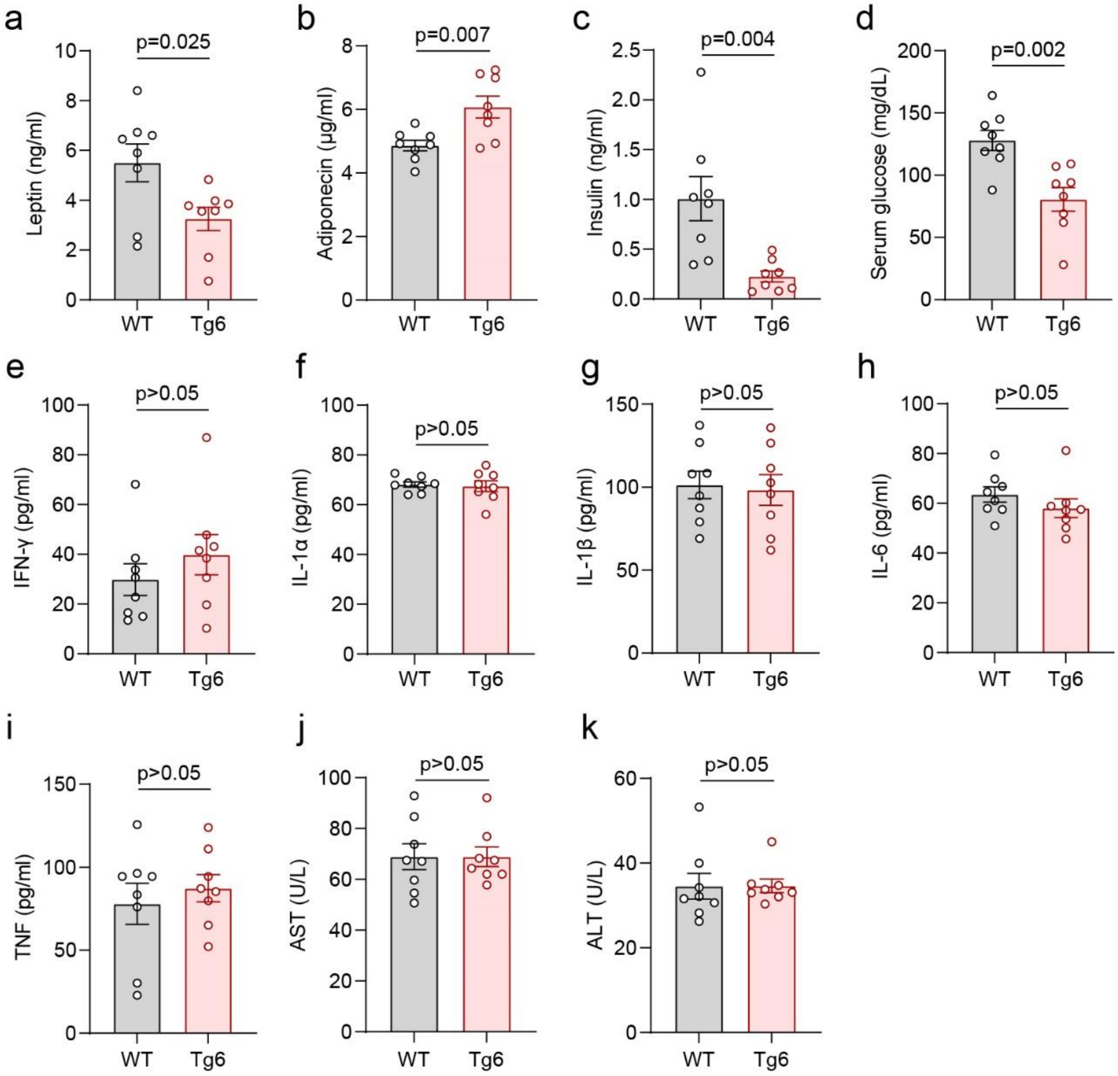

Supplementary Figure 3. Measurements of metabolic parameters, serum glucose, inflammatory cytokines and liver function in Tg6-mice and WT-mice. a-k, ELISA assays were used to measure serum levels of leptin (**a**), adiponectin (**b**), insulin (**c**); inflammatory cytokines, IFN-γ (**e**), IL-1α (**f**), IL-1β (**g**), IL-6 (**h**), and TNF (**i**); liver function, AST (**j**) and ALT (**k**) in WT (grey) and Tg6 (red) mice. Glucometer was used to determine serum glucose (**d**). WT: n = 8, Tg6: n = 8 indicates the number of mice in each group. p-values are indicated. Two-tailed unpaired t-test. Source data are provided as a Source Data file.

Supplementary Figure 4

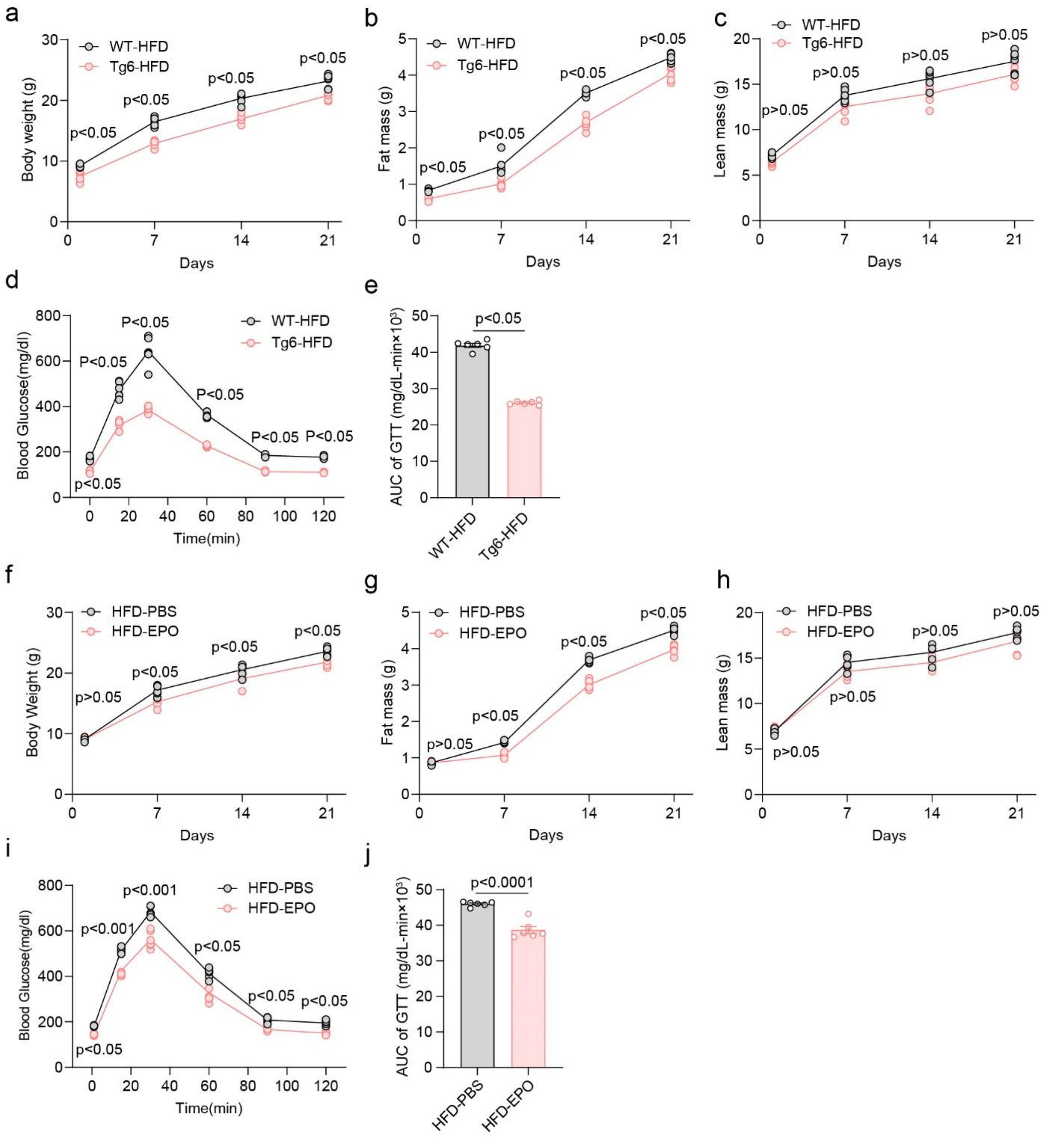

Supplementary Figure 4. Body composition and glucose tolerance after high fat diet (HFD) feeding of WT-mice treated with EPO or saline, and Tg6-mice. a-e, Tg6-mice (red) and WT littermate control mice (black) were fed HFD for three weeks and body weight (**a**), fat mass (**b**), and lean mass (**c**) were monitored, and glucose tolerance (GTT) (**d**) and area under the curve (AUC of GTT) (**e**) were determined. p-values are indicated. **f-j,** WT-mice were fed HFD for three weeks were treated with EPO (red), or

phosphate buffered saline (PBS, black) and body weight (**f**), fat mass (**g**), and lean mass (**h**) were monitored, and glucose tolerance (GTT) (**i**) and area under the curve (AUC of GTT) (**j**) were determined. a-j, n = 6 indicates the number of mice in each group, p-values are indicated. t-test, Two-way ANOVA with Bonferroni's multiple comparisons test. Source data are provided as a Source Data file.

Supplementary Figure 5

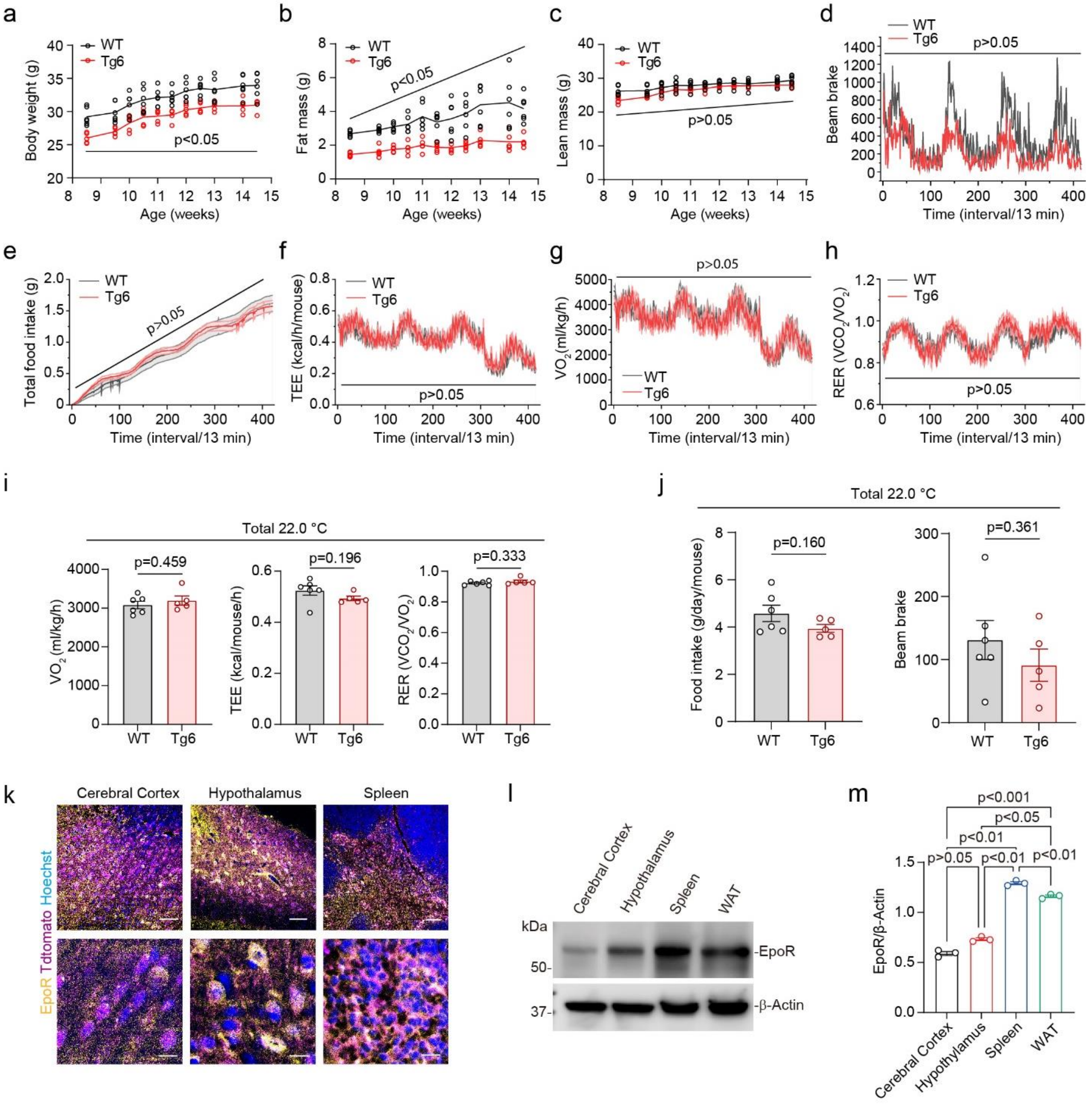

Supplementary Figure 5. Metabolic measurements by indirect calorimetry did not identify significant differences between male WT-mice and male Tg6-mice. a-c, Body weight (**a**), fat mass (**b**) and lean mass (**c**) were determined for Tg6-mice (red) and their littermate control (WT, black). **d,** Mouse activity with time expressed in intervals of 13 minutes for up to 4 days was recorded for Tg6 (red) and control

(black) mice. **e,** Food intake was also recorded for Tg6 (red) and control (black) mice. **f-h,** TEE (**f**) Volume of O_2 (**g**), RER (**h**) were monitored for Tg6 (red) and control (black) mice with time expressed in intervals of 13 minutes. **i-j,** Volume of O_2, TEE, and RER of mice at 22°C (**i**) and food intake and mouse activity (**j**) at 22°C in Tg6 (red) and control (black) mice were recorded. **k**, Immunofluorescence staining for EpoR and tdTomato, and Hoechst from the cerebral cortex, hypothalamus, and spleen. Scale bar: upper, 50 μm, lower, 10 μm. **l**, Immunoblot analysis was conducted to assess the expression levels of EpoR across various organs, with β-Actin serving as the loading control. **m**, Quantification of figure l. n = 3, One-way ANOVA with Bonferroni's multiple comparison test. **a-h**, WT, n = 7 mice; Tg6, n = 5 mice. **i-j**, WT, n = 6 mice; Tg6, n = 5 mice. p-values are indicated. Two-tailed unpaired t-test, One-way or Two-way ANOVA with Bonferroni's multiple comparisons test. Source data are provided as a Source Data file.

Supplementary Figure 6

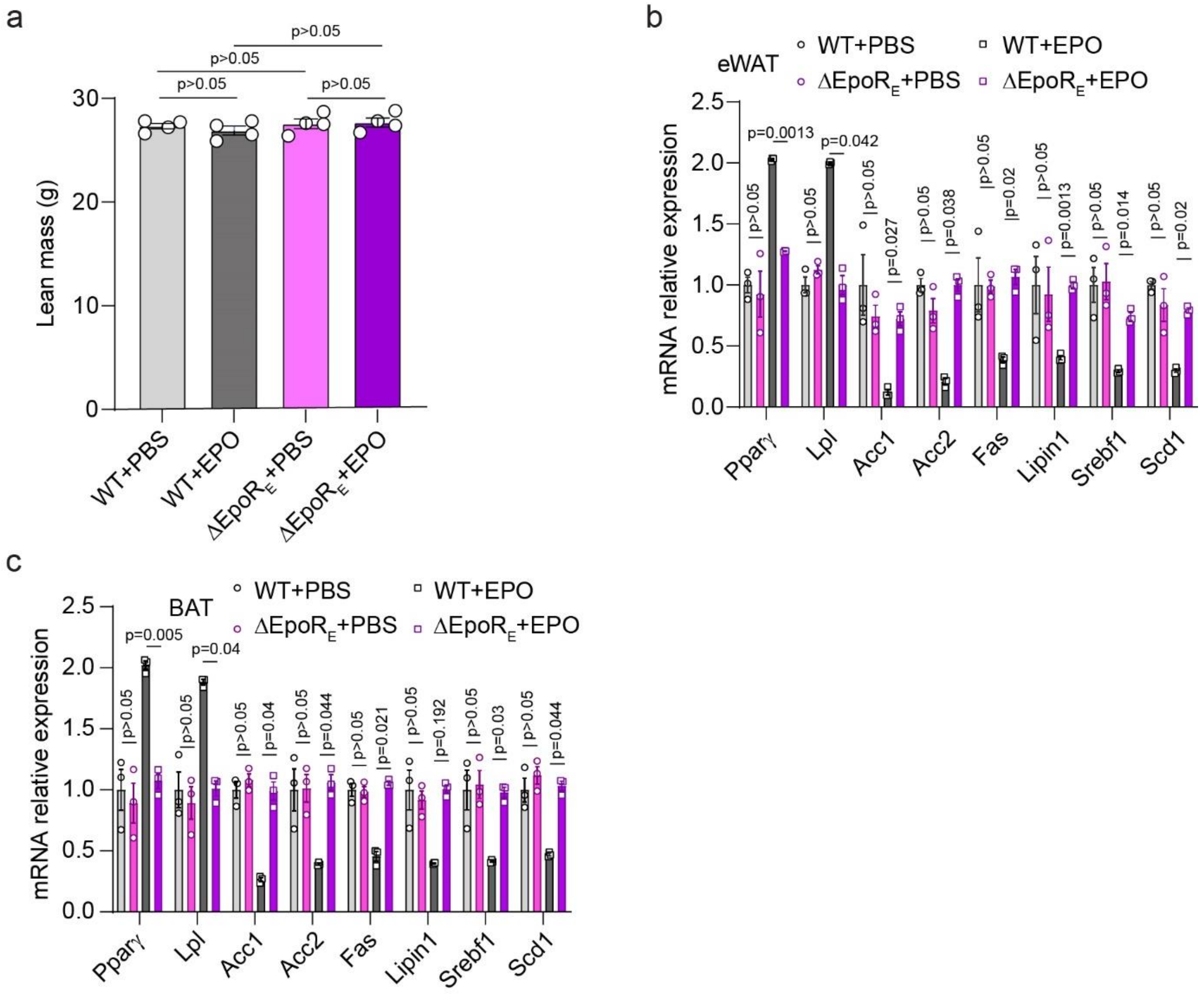

Supplementary Figure 6. Expression of lipid metabolism-associated genes in mice treated with EPO or PBS. a, Lean mass was measured in WT-mice treated with PBS (light grey) or EPO (dark grey) and $\Delta EpoR_E$ mice treated with PBS (light purple) or EPO (purple). **b-c,** Tissues were harvested and assessed for gene expression determined by real-time quantitative PCR (qPCR) for *Pparγ, Lpl, Acc1, Acc2, Fas, Lipin1, Srebf1, and Scd1* for eWAT (**b**), and BAT (**c**). Colors indicated for **b** and **c** are light grey (circles) for WT+PBS, dark grey (squares) for WT+EPO, light purple (circles) for $\Delta EpoR_E$+PBS and purple (squares) for $\Delta EpoR_E$+EPO. a, n=4 mice per group; b-c: n = 3 mice per group. p-values are indicated. One way or Two-way ANOVA with Bonferroni's multiple comparisons test. Source data are provided as a Source Data file.

Supplementary Figure 7

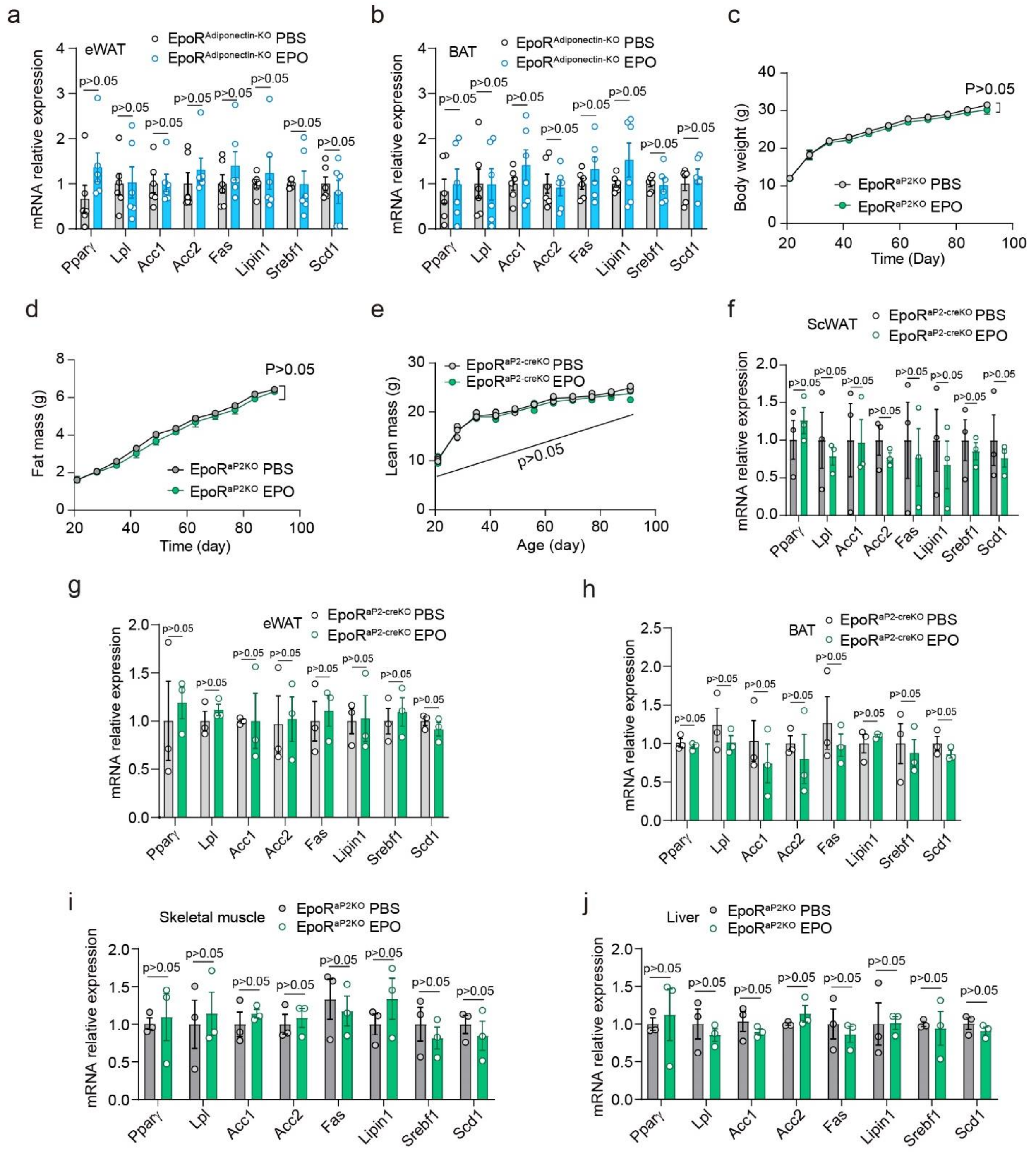

Supplementary Figure 7. Ablation of EpoR in adipocytes diminished the EPO effect on lipid metabolism. a-b, EpoR$^{Adiponectin\text{-}KO}$ mice were generated and gene expression for lipid metabolism genes, *Pparγ, Lpl*, *Acc1*, *Acc2, Fas*, *Lipin1*, *Srebf1*, and *Scd1* were quantified by qRT-PCR from eWAT (**a**) and BAT (**b**) from PBS (gray) or EPO treated (blue) EpoR$^{Adiponectin\text{-}KO}$ mice. **c-j,** EpoR$^{aP2\text{-}creKO}$ mice were

generated by crossing aP2-Cre mice with $EpoR^{floxp/floxp}$ mice. Body weight (**c**), fat mass (**d**), and lean mass (**e**) were monitored in PBS (gray) or EPO treated $EpoR^{aP2KO}$ (green) mice. Expression of lipid metabolism genes, *Pparγ*, *Lpl*, *Acc1*, *Acc2*, *Fas*, *Lipin1*, *Srebf1*, and *Scd1*, were quantified by qRT-PCR for ScWAT (**f**), eWAT (**g**), BAT (**h**), skeletal muscle (**i**), and liver (**j**) from PBS (gray) or EPO treated (green) $EpoR^{aP2KO}$ mice. No significant differences were observed between PBS and EPO treatment in $EpoR^{Adiponectin\text{-}KO}$ or $EpoR^{aP2KO}$ mice. a-b, n = 6 mice per group; c-j, n = 3 mice per group; p-values are indicated. Two-way ANOVA with Bonferroni's multiple comparisons test. Source data are provided as a Source Data file.

Supplementary Figure 8

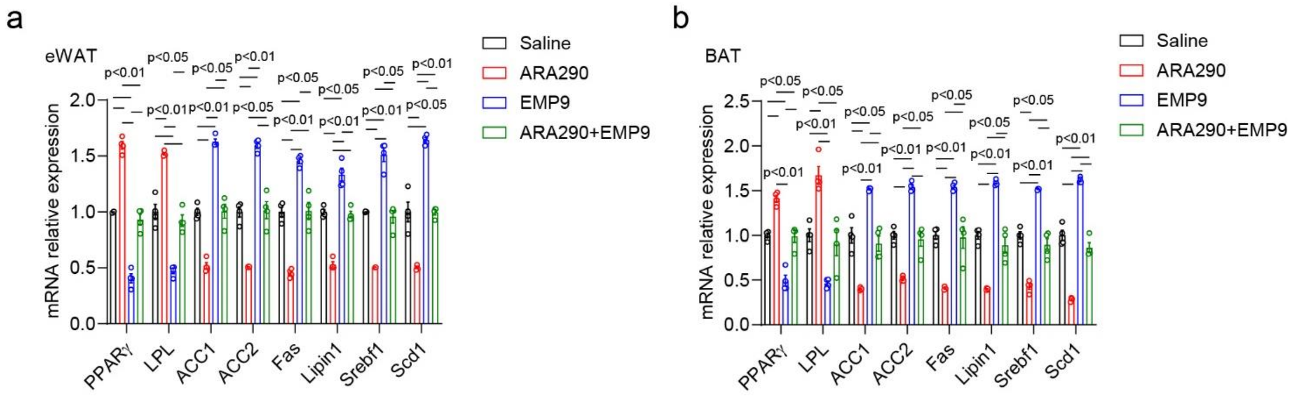

Supplementary Figure 8. Non-erythropoietic EpoR agonist ARA290 mimics the EPO effect on lipid metabolism genes expression. a-b, Expression of lipid metabolism genes in the eWAT (**a**) and BAT (**b**) was assayed with qRT-PCR in WT mice fed normal diet and treated with ARA290, EMP9, or ARA290+EMP9, saline treated group was used as control. a, b, n= 4 mice per group. p-values are indicated. Two-way ANOVA with Tukey's multiple comparisons test. Source data are provided as a Source Data file.

Supplementary Figure 9

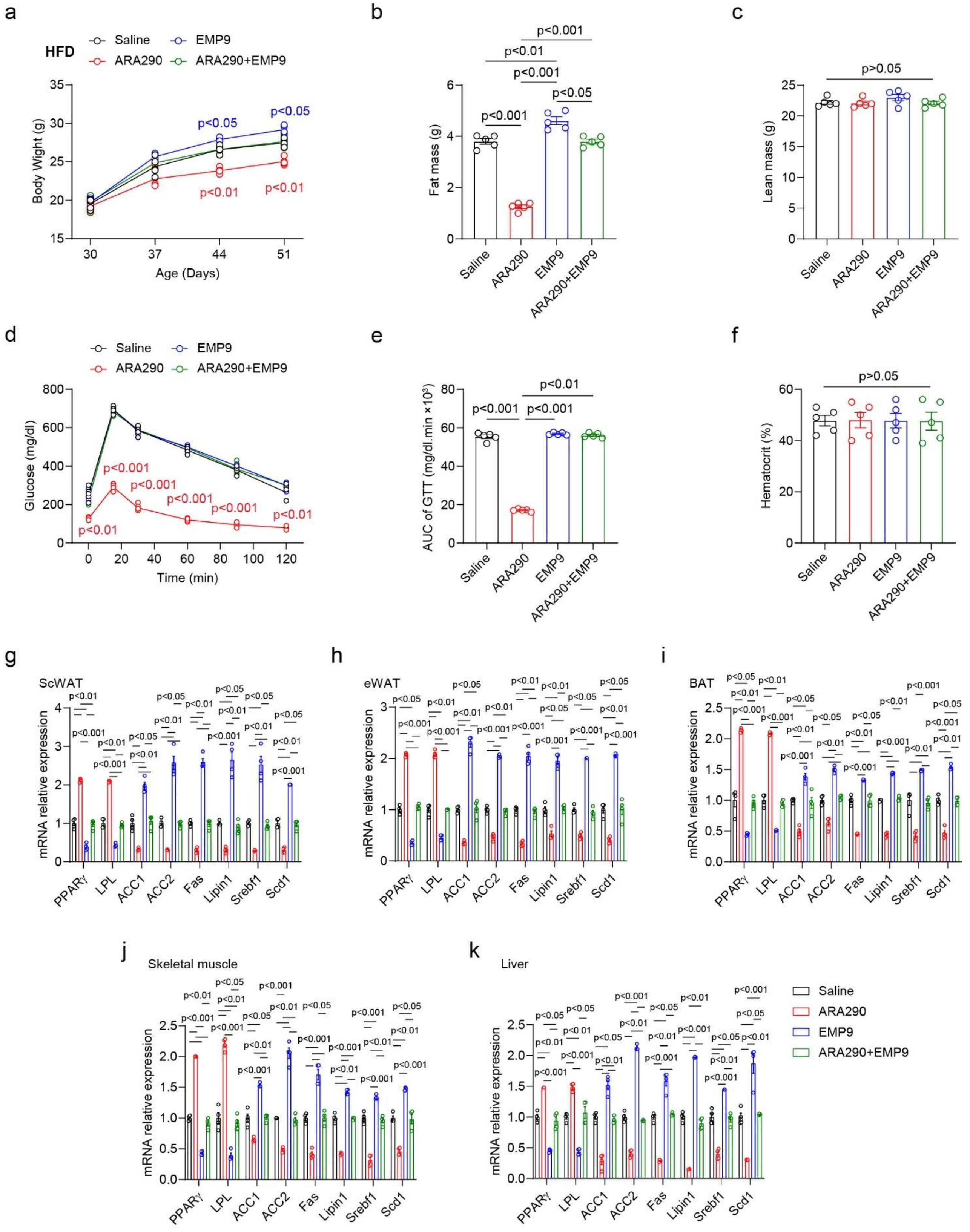

Supplementary Figure 9. Non-erythropoietic EpoR agonist ARA290 treatment in WT mice fed HFD decreased fat mass and increased glucose tolerance, without increasing EPO stimulated erythropoiesis. (**a-f**) WT mice were fed on high fat diet (HFD) and treated with Saline (black), ARA290 (red), EMP9 (blue), or ARA290+EMP9 (green) for three weeks and body weight (**a**), fat mass (**b**), lean mass (**c**), glucose tolerance (GTT) (**d**) and area under the curve (AUC of GTT) (**e**), and hematocrit (**f**) were monitored. a-f, n = 5, p-values are indicated. Not significant is not shown in the figure. One-way ANOVA with Tukey's multiple comparisons test. **g-k**, Expression of lipid metabolism genes in the ScWAT (**g**), eWAT (**h**), BAT (**i**), Skeletal muscle (**j**), and Liver (**k**) was assayed with qRT-PCR in WT mice feed with HFD food and treated with ARA290, EMP9, or ARA290+EMP9, saline treated group was used as control. g-k, n= 4 mice per group. p-values are indicated. Two-way ANOVA with Tukey's multiple comparisons test. Source data are provided as a Source Data file.

Supplementary Figure 10

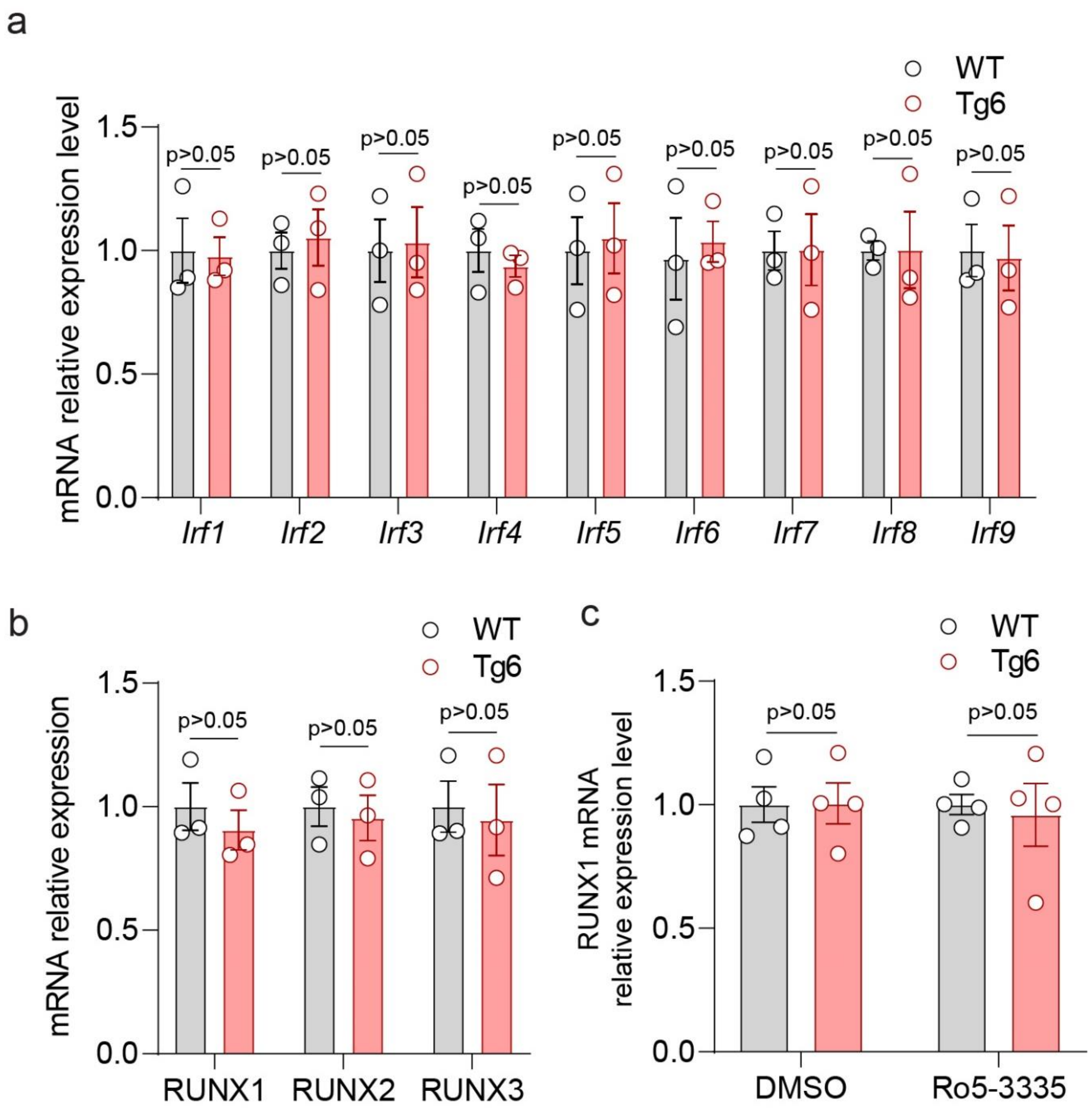

Supplementary Figure 10. Chronic high transgenic EPO expression in young Tg6-mice has no effect on Irf family members or RUNX1 mRNA levels in scWAT. **a**, Gene expression for Irf family members was determined by qPCR for scWAT from WT-mice (grey) and Tg6-mice (red) (n = 3 mice per group). **b**, RUNX family mRNA relative expression levels were measured by qPCR in scWAT of WT-mice (grey) and Tg6-mice (red); n = 3 mice per group. **c**, RUNX1 mRNA relative expression in scWAT was determined for RUNX1-inhibitor Ro5-3335 or DMSO vehicle treated WT-mice (grey) and Tg6-mice

(red); n = 4 mice per group, p-values are indicated. Two-way ANOVA with Bonferroni's multiple comparisons test. Source data are provided as a Source Data file.

Supplementary Figure 11

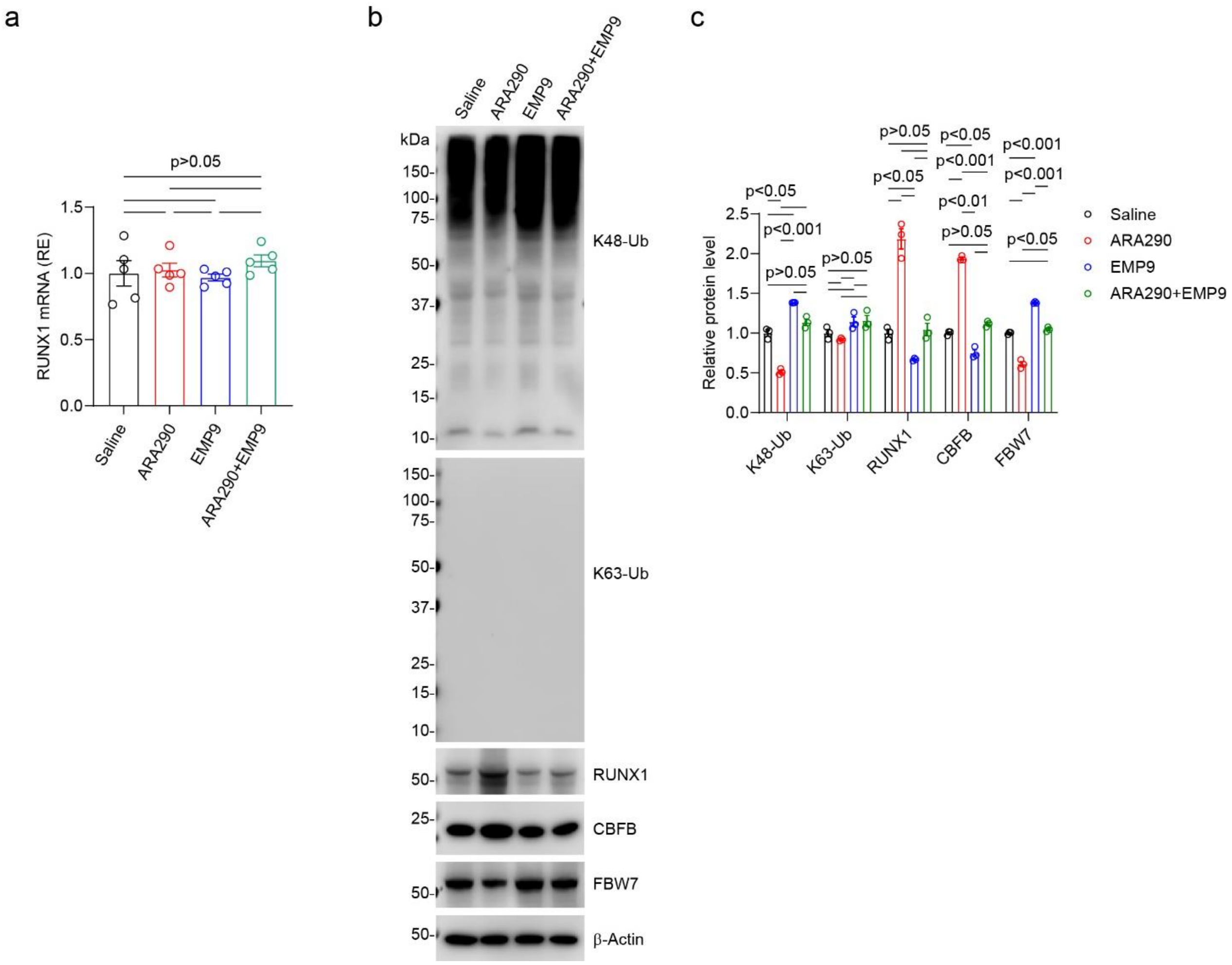

Supplementary Figure 11. Non-erythropoietic EPOR agonist ARA290 mimics the EPO effect on RUNX1 and CBFb protein degradation by FBW7 in ScWAT tissue of mice. a, Gene expression for RUNX1 was determined by qPCR for scWAT from different mice treated as indicated. (n =5 mice per group group, One-way ANOVA with Tukey's multiple comparisons test). **b,c**, K48 Ubiquitin, K63 Ubiquitin, RUNX1, CBFβ and FBW7 protein expression were determined by Western blotting with β-Actin as control (left) for scWAT from mice treated as indicated (**b**), and relative protein level were quantified by ImageJ (**c**). (n = 3 mice per group, a representative sample is shown; One-way ANOVA with Tukey's multiple comparisons test). Source data are provided as a Source Data file.

Supplementary Figure 12

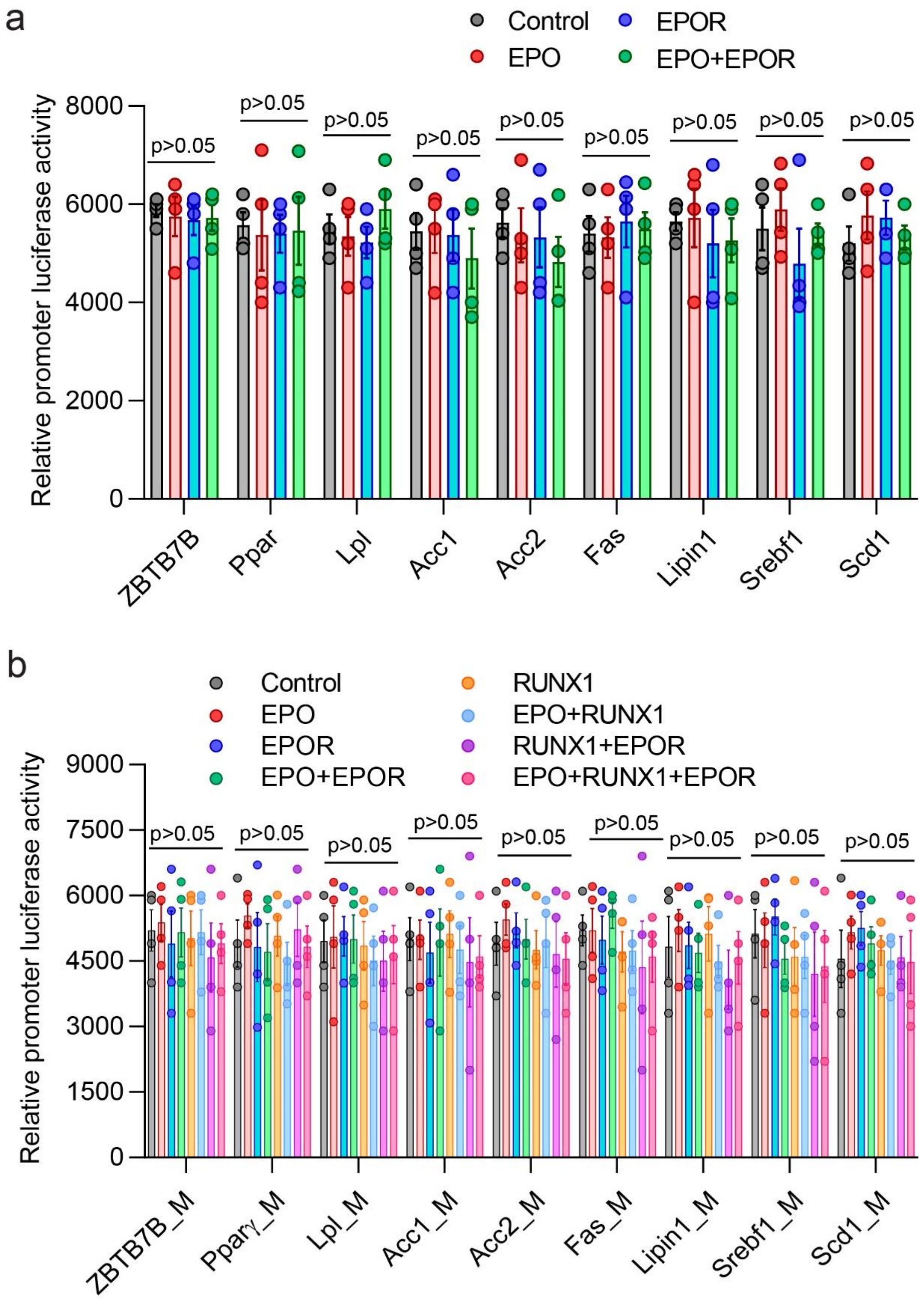

Supplementary Figure 12. Reporter gene assays for luciferase reporter plasmids containing RUNX1 binding region identified in genes associated with lipid metabolism and with the RUNX1 binding site mutated (Supplementary Table 3). a, Luciferase assay for transcription activity of the RUNX1 binding region from the promoters of *Ppar*γ, *Lpl*, *Acc1*, *Acc2*, *Fas*, *Lipin1*, *Srebf1,* and *Scd1* genes and ZBTB7B control (RUNX1 silencer activity) inserted upstream of a minimal promoter reporter gene construct (pNL3.1[Nluc/minP]) was determined in HEK293T cells. No significant differences were observed for each luciferase reporter without or with co-transfection of EpoR expression plasmid and without or with EPO treatment (control (grey), EPO treatment (red), EpoR expression plasmid (blue) and EpoR expression plasmid plus EPO treatment (green, EPO+EPOR). **b**, Reporter gene activity was assessed in HEK293T cells transfected with each reporter gene construct with the RUNX1 binding site mutated from the promoters of *Ppar*γ, *Lpl*, *Acc1*, *Acc2*, *Fas*, *Lipin1*, *Srebf1*, and *Scd1* genes and ZBTB7B control without (grey, control) and with (orange, RUNX1) co-transfection of RUNX1 expression plasmid. Additional activation of the EPO-EpoR axis was assessed using EPO treatment (red, EPO; light blue, EPO+RUNX1) and with and without co-transfection of EpoR expression plasmid (blue, EPOR; purple, RUNX1+EPOR; green, EPO+EPOR; pink, EPO+RUNX1+EPOR). n = 4 replicates in each group, the result shown in figure represent one of three independent experiments. p-values are indicated. Two-way ANOVA with Bonferroni's multiple comparisons test. Source data are provided as a Source Data file.

Supplementary Figure 13

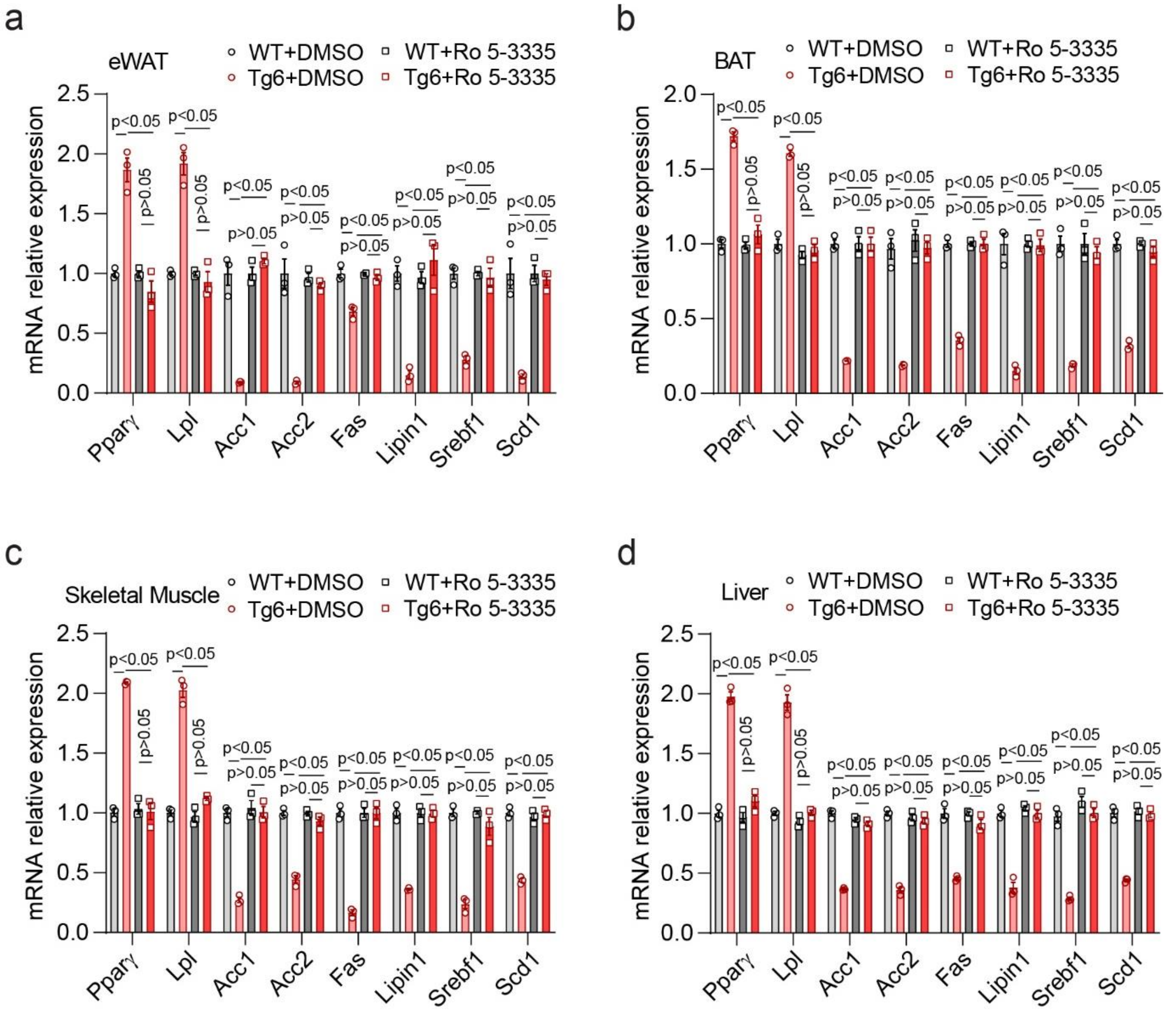

Supplementary Figure 13. Expression of lipid metabolism-associated genes in Tg6-mice with high transgenic EPO-treated with RUNX1-inhibitor, Ro5-3335. a-d, Tg6-mice were treated with Ro5-3335 (square, red) or DMSO vehicle (circle, light red) and compared with WT-mice treated with Ro5-3335 (square, dark grey) or DMSO vehicle (circle, light grey). Tissues were harvested and gene expression for lipid metabolism genes, *Pparγ*, *Lpl*, *Acc1*, *Acc2*, *Fas*, *Lipin1*, *Srebf1*, and *Scd1* was determined by real-time quantitative PCR (qPCR) for eWAT (**a**), BAT (**b**), skeletal muscle (**c**), and liver (**d**). n = 3 mice per group, p-values are indicated. Two-way ANOVA with Bonferroni's multiple comparisons test. Source data are provided as a Source Data file.

Supplementary Figure 14

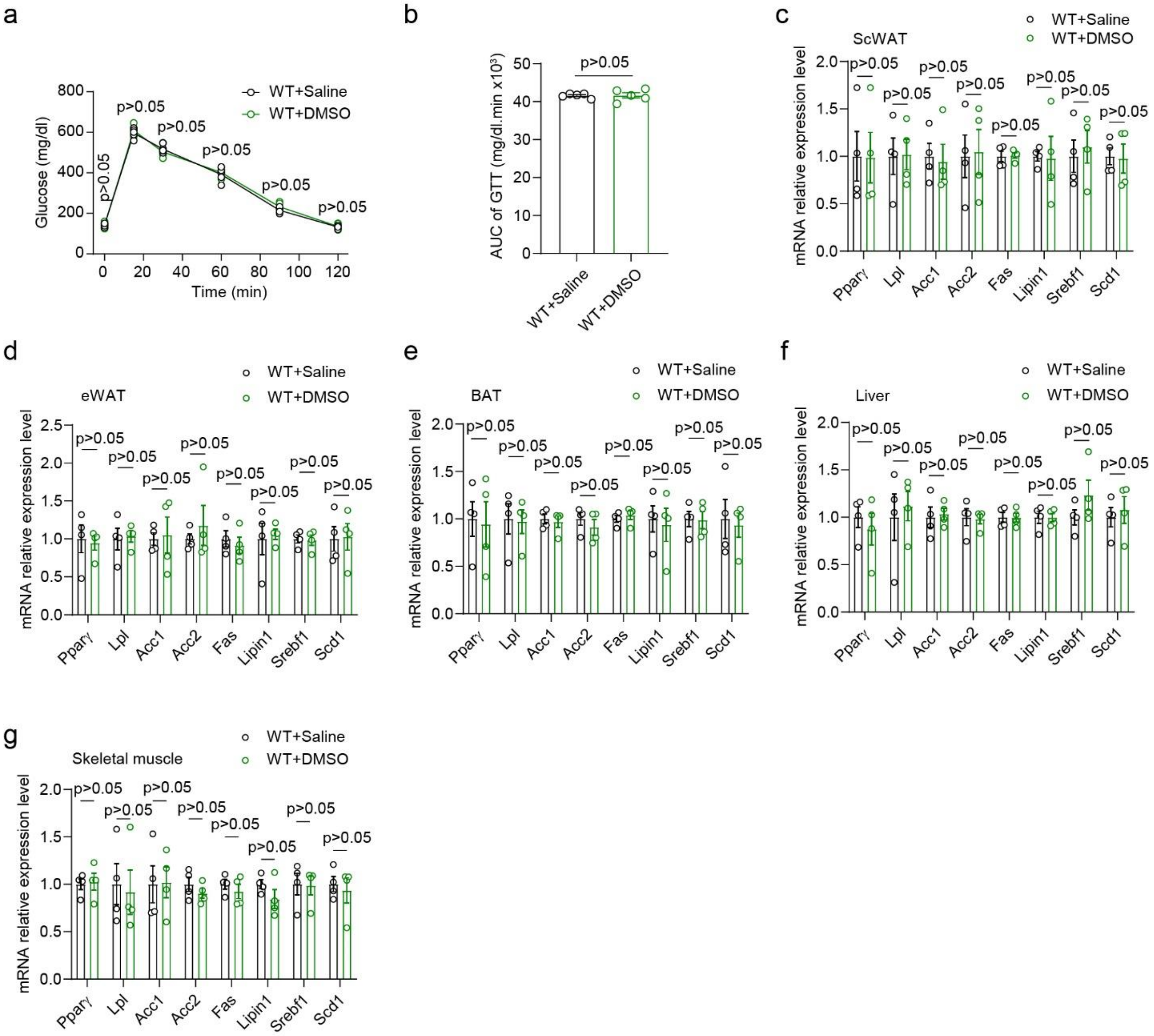

Supplementary Figure 14. Comparison between Saline and DMSO vehicle control. a-b, WT-mice were treated with saline (100 μl) or DMSO (0.75 μl DMSO was diluted in 100 μl Saline) for three weeks, and glucose tolerance (GTT) (**a**) and area under the curve (AUC of GTT) (**b**) were determined. **c-h**, Expression of lipid metabolism genes, *Pparγ*, *Lpl*, *Acc1*, *Acc2*, *Fas*, *Lipin1*, *Srebf1*, and *Scd1*, were quantified by qRT-PCR for ScWAT (**c**), eWAT (**d**), BAT (**e**), liver(**f**), and skeletal muscle (**g**) from saline (black) or DMSO treated (green) in WT mice. No significant differences were observed between saline and DMSO treatment in WT mice. a-b, n=5, c-g, n = 4 mice per group; p-values are indicated ($p>0.05$ means no significant difference). Two-way ANOVA with Bonferroni's multiple comparisons test. Source data are provided as a Source Data file.

Supplementary Figure 15

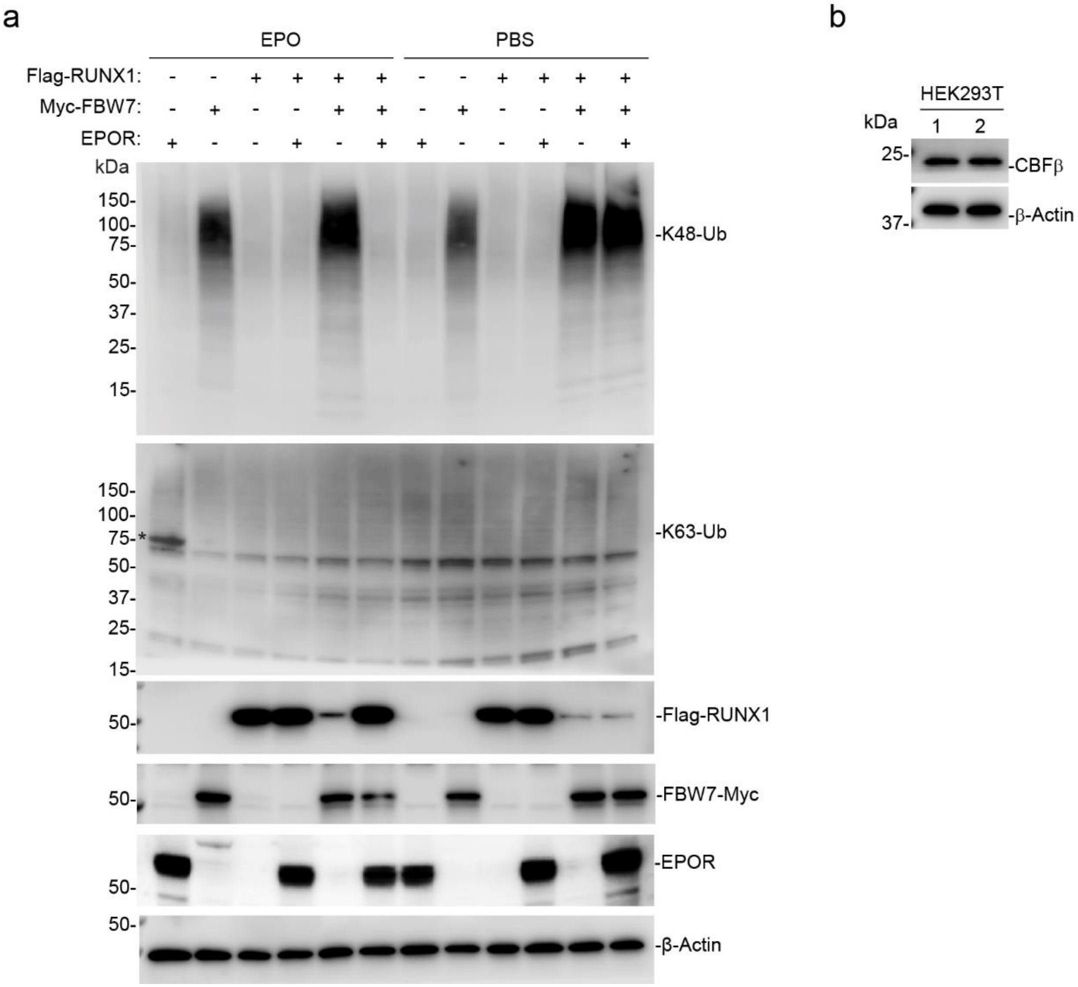

Supplementary Figure 15. Assessment of relative protein stability of RUNX1 by activation of the EPO-EpoR axis without over-expression of CBFβ. a. HEK293T cells were transfected with expression plasmids for FBW7 (FBW7-Myc), and/or RUNX1 (Flag-RUNX1), and/or EpoR and were treated with EPO (3 U/ml) or PBS for 24 h. The ubiquitylation of K48-Ub and K63-Ub, and protein levels of Flag-RUNX1, FBW7-Myc, and EpoR were determined by Western blotting with β-actin as control. * Indicated the non-specific band. **b.** Western blot analysis revealed the endogenous expression of CBFβ in HEK293T cells. Source data are provided as a Source Data file.

Supplementary Figure 16

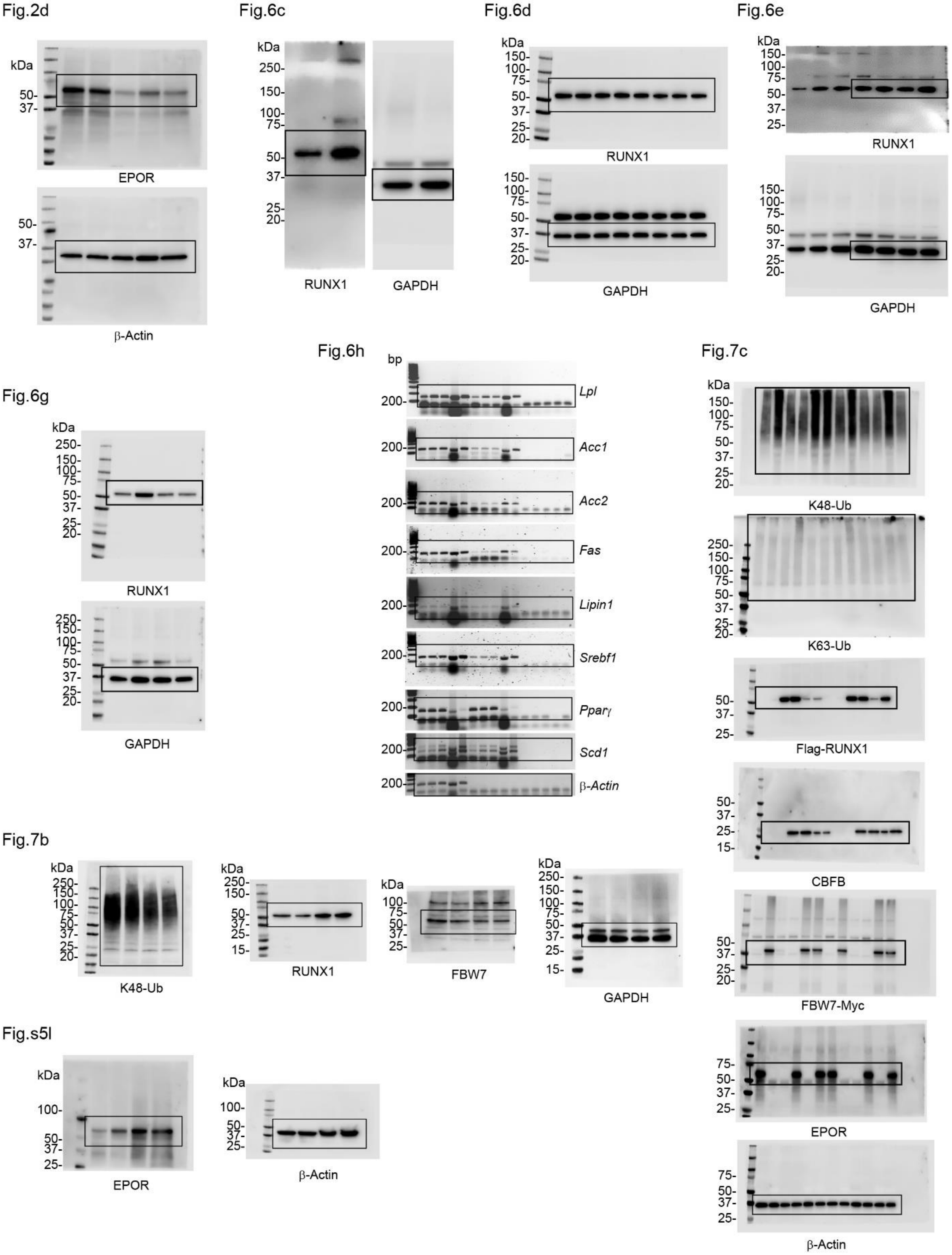

Fig.s11b

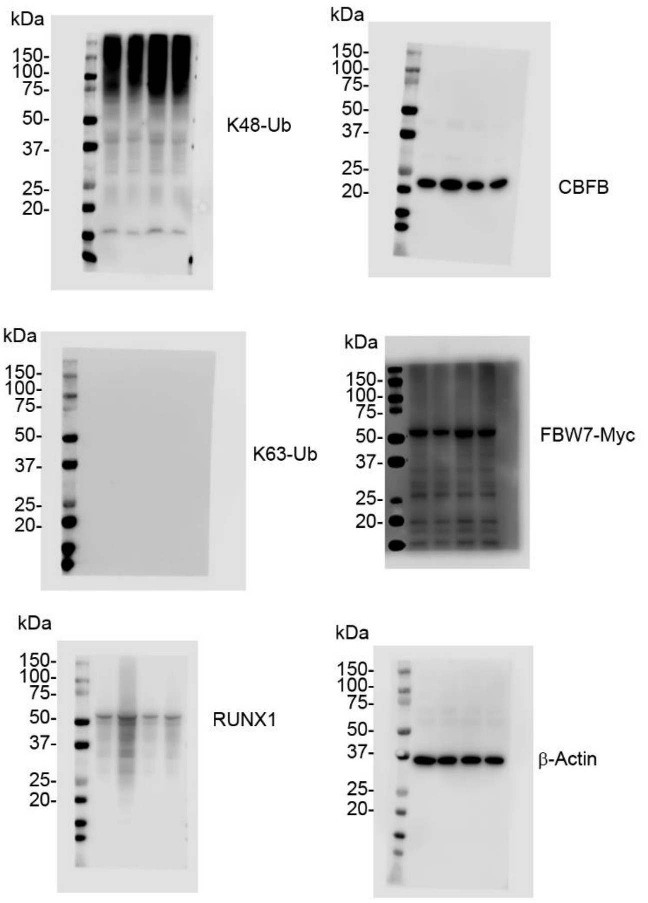

Fig.s15a

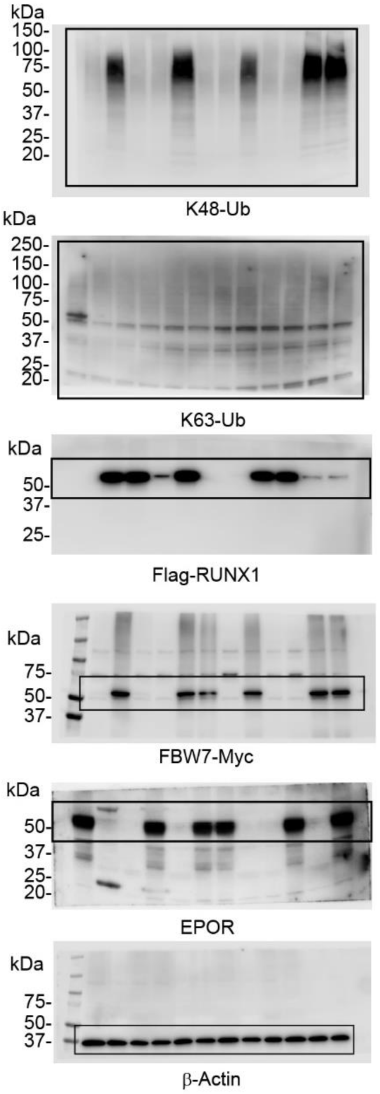

Fig.s15b

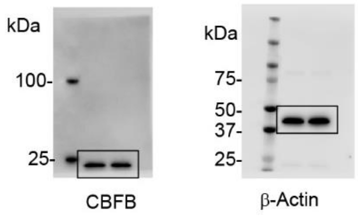

Supplementary Figure 16. Original results from Western blotting and PCR for ChIP assay. Shown are images from Western blotting for figures indicated (Fig.2d, Fig.5c, Fig.5d, Fig.5e, Fig.5g, Fig.6b, Fig.6c, Fig.s5l, S11b and Fig.s15a, b). Also shown are images for PCR results from ChIP assay for Fig.5h.

Supplementary Tables

Supplementary Table 1: Antibodies used in this study

Antibodies	Concentration for Western Blotting/IF	Catalog number	Sources
Anti-Erythropoietin R Antibody	1:2000	AF1390	Novus Biologicals
Anti-RFP antibody	1:1000	600-401-379	ROCKLAND
Anti-RUNX1 (middle) Polyclonal Antibody	1:2000	25315-1-AP	Thermo Fisher Scientific
Anti-UCP1 Antibody (536435)	10 µg/mL	MAB6158	Novus Biologicals
Anti- K48-linkage Specific Polyubiquitin Antibody	1:1000	4289	Cell signaling
Anti-K63-linkage Specific Polyubiquitin (D7A11) Rabbit mAb	1:1000	5621	Cell signaling
Anti-FBXW7 Polyclonal Antibody	1:1000	40-1500	Thermo Fisher Scientific
DYKDDDDK Tag (D6W5B) Rabbit mAb (Binds to same epitope as Sigma's Anti-FLAG® M2 Antibody)	1:1000	14793	Cell signaling
Myc-Tag (9B11) Mouse mAb	1:1000	2276	Cell signaling
Rabbit anti-CBF-beta (isoform 1) Antibody	1:1000	A303-549A	FORTIS LIFE SCIENCES
Rabbit anti-β-Actin Antibody	1:1000	4967	Cell signaling
Mouse anti-GAPDH Antibody (1D4)	1:3000	MA1-16757	Thermo Fisher Scientific
Goat anti-Mouse IgG (H+L) Secondary Antibody,HRP	1:3000	31430	Thermo Fisher Scientific
Goat anti-Rabbit IgG (H+L) Secondary Antibody, HRP	1:3000	31460	Thermo Fisher Scientific
Rabbit anti-Goat IgG (H+L) Secondary Antibody, HRP	1:3000	31402	Thermo Fisher Scientific
Donkey anti-Goat IgG (H+L) Cross-Adsorbed Secondary Antibody, Alexa Fluor™ 488	1:200	A-11055	Thermo Fisher Scientific

Donkey anti-Goat IgG (H+L) Cross-Adsorbed Secondary Antibody, Alexa Fluor™ 568	1:200	A-11057	Thermo Fisher Scientific
Goat anti-Mouse IgG (H+L) Cross-Adsorbed Secondary Antibody, Alexa Fluor™ 488,	1:200	A-11001	Thermo Fisher Scientific
Goat anti-Mouse IgG (H+L) Secondary Antibody Alexa Fluor™ 568	1:200	A-11004	Thermo Fisher Scientific
Goat anti-Rabbit IgG (H+L) Cross-Adsorbed Secondary Antibody, Alexa Fluor™ 568	1:200	A-11011	Thermo Fisher Scientific

Supplementary Table 2: Primers used in this study

Primer name	Primer sequence
*mEpoR forward	GCTCCGGGATGGACTTCA
*mEpoR reverse	GAGCCTGGTGCAGGCTACAT
*mEpoR probe	CATACCAGCTCGAGGGTGAGTCACGAAAG
*16S forward	GATCGAGCCGCGCACG
*16S reverse	CAAATCGCTCCTTGCCCA
*16S probe	CTGCAGTACAAGTTACTGGAGCCTGTTTTGCT
Pparγ forward	CAGGAAAGACAACAGACAAATCA
Pparγ reverse	GGGGGTGATATGTTTGAACTTG
LPL forward	CAAAACAACCAGGCCTTCGA
LPL reverse	AGCAATTCCCCGATGTCCA
Acc1 forward	GATGATCAAGGCCAGCTTGT
Acc1 reverse	CAGGCTACCATGCCAATCTC
Acc2 forward	GCACGAGATTGCTTTCCTAG
Acc2 reverse	GCTTCCGCTCCAGGGTAGAGT
Fas forward	CTTGGGTGCCGATTACAACC
Fas reverse	GCCCTCCCGTACACTCACTC
Lipin1 forward	TCACTACCCAGTACCAGGGC
Lipin1 reverse	TGAGTCCAATCCTTTCCCAG
Srebf1 forward	GGAGCCATGGATTGCACATT
Srebf1 reverse	AGGAAGGCTTCCAGAGAGGA
Scd1 forward	CTACAAGCCTGGCCTCCTGC
Scd1 reverse	GGCACCCAGGGAAACCAGGA
Ucp1 forward	GGCCCTTGTAAACAACAAAATAC
Ucp1 reverse	GGCAACAAGAGCTGACAGTAAAT
Pgc1α forward	CAATGAATGCAGCGGTCTTA
Pgc1α reverse	GTGTGAGGAGGGTCATCGTT
Cidea forward	ATGTCCCAGTCTGCAAGCAA
Cidea reverse	TGTGCATCGGATGTCGTAGG
Prdm16 forward	CAACTATCAGCGCGTTTGCG
Prdm16 reverse	GAGGCCTTCTCGGTCTTCAC
Runx1 forward	TTTCGCAGAGCGGTGAAAGA
Runx1 reverse	GCACTGTGGATATGAAGGAA
GAPDH forward	TGAACGGGAAGCTCACTGG
GAPDH reverse	TCCACCACCCTGTTGCTGTA
18S forward	AGTCCCTGCCCTTTGTACACA
18S reverse	CGATCCGAGGGCCTCACTA
March1 forward	ATGGAGACCAAGCTCAAGCC
March1 reverse	GATTTCCTCCGCTGTCCGAT
March5 forward	ATGCAGGTTCACCATTCTCTATT
March5 reverse	AATAACGGGTCAGCTCGCTC
TRIM5 forward	ATAAAACGAGGGCAAGCACC
TRIM5 reverse	TTTGTTCCCTGGCGATGGTT
TRIM11 forward	ACTGGAGCACCTACGGAAAC
TRIM11 reverse	TCCTTAATGTCCTGCAGTAAACCCA
TRIM21 forward	AAGCTGAGGACCCTGGTTAGA
TRIM21 reverse	TGGAATCTGAATGACAGGAGCC
TRIM23 forward	GCGTGTGCCTCTAGCTGATA

TRIM23 reverse	TACCTGGGACCTTGTGACCT
TRIM26 forward	CTGCTTGCTCAGGACCTACC
TRIM26 reverse	AGACCTGCTTCTGGTCATGC
TRIM27 forward	ATTTCCGTGCCCCTACAGTG
TRIM27 reverse	TGGGTCTCCAAATCCAAATCCT
TRIM32 forward	CGGGCGGTCAGGAATCT
TRIM32 reverse	AGATGGTATGGCCACAGTGC
TRIM35 forward	CCAGCCTCAAGTTAGTGCCA
TRIM35 reverse	AGCATGAGGTGACCAACCAG
Mylip forward	GGTAAAGCGAGGCCGAAACA
Mylip reverse	GTTGAAAAACCCACCCAGGC
Mkrn1 forward	CAGCCGCTTTTGTATGCCTT
Mkrn1 reverse	TCTCGCAAAGAAGAACGCCT
COP1 forward	CAACGACTTCGTCTGTCCCA
COP1 reverse	GCCCATTGGTGCTACTCACT
Peli3 forward	CACACAGAGAGCCCCAGAAC
Peli3 reverse	AAGACAGGATGCAAGAGCCC
FBXW7 forward	GGTGAAGGTGTGGGATCCAG
FBXW7 reverse	CTGGTGTCCTGTTAGCGTGT
# Lpl-P-F	5’-GACAGACAGAATTTCCTTAC-3’
# Lpl-P-R	5’-GGCCAGGTCTGTTTTGATATG-3’
# Fas-P-F	5’-GACAATGCTGCAGGATGTCC-3’
# Fas-P-R	5’-CTCGCTCAGCTTCTGTTTCTA-3’
# ACC1-P-F	5’-TCAGAAACCAGTCCAAGGTA-3’
# ACC1-P-R	5’-GGTGAATACCACCATACCAGC-3’
# ACC2-P-F	5’-CCATGTGGCCTTTTCAGTGT-3’
# ACC2-P-R	5’-CATAGATATCACGCACACCCAA-3’
# Lipin1-P-F	5’-TCATAGAGTAAACCATAGAG-3’
# Lipin1-P-R	5’-CTCTTACTTCCGTGCTGTGG-3
# Srebf1-P-F	5’-GCACATGGGCTTCCCTTTCT-3’
# Srebf1-P-R	5’-CCCTAGATGAGGGTTGGTCT-3’
# Pparg-P-F	5’-CACTGAAGGCAGTAGAATTA-3’
# Pparg-P-R	5’- GTCTTGTATGATTGGTTTCC-3’
# Scd1-P-F	5’-CAGAAGAAAGAAAGGAACTA-3’
# Scd1-P-R	5’-GCCAAATGCAGCCTGGTCTA-3’

*indicates primers and probes for Taqman PCR; # indicates primers for Chip

Supplementary Tables 3: Gene specific RUNX1 binding region cloned into reporter gene

Gene	Identified RUNX1 binding site with flanking Xho1/HindIII sites
Acc1 (XhoI/HindIII)	CTCGAGAGATGCACACAGATCAGAAACCAGTCCAAGGTATATTTTG<br>GCAACCCGACACGGCAACTGTGTGGTTGCCAACCACAACACTCAGA<br>CCCCCCCCCTCGCTCACAGAATGTGAAGTGCAGCCTCCTTACAGAA<br>CCTGGAACTTGCTGGTATGGTGGTATTCACCTTTAATCCTATGCTAA<br>AAAGCTT
Acc1 mutated RUNX1 (XhoI/HindIII)	CTCGAGAGATGCACACAGATCAGAAACCAGTCCAAGGTATATTTTG<br>GCAACCCGACACGGCTACGGACCTAGCGTTAGCTAGCTAGCGCTAG<br>AAATCGCGCGCTTGAGAGCGCGAAATTCATCAGCCTCCTTACAGAA<br>CCTGGAACTTGCTGGTATGGTGGTATTCACCTTTAATCCTATGCTAA<br>AAAGCTT
Acc2 (XhoI/HindIII)	CTCGAGTCCATGTGGCCTTTTCAGTGTTTATATAGTGTGTGCGCAAA<br>TGCGCGTATGTGTGTGTGTGTGTGTGTGTGTGTGTGTGTGTGTGTGT<br>GTGTTTCAGGAGTGGGGGTGGGAGGAGTCATCGTCGTCAGATGTGT<br>CTAACGATTTTTCCTTTGGGTGTGCGTGATATCTATGCACACAGGTA<br>AGCTT
Acc2 mutated RUNX1 (XhoI/HindIII)	CTCGAGTCCATGTGGCCTTTTCAGTGTTTATATAGTGTGTGCGCAAA<br>GTACGTACGGATCTCAGAGAGTCGATACGAATCGATCGATCGAGAA<br>ATCGCGCGTACGGAAGGGGTGGGAGGAGTCATCGTCGTCAGATGT<br>GTCTAACGATTTTTCCTTTGGGTGTGCGTGATATCTATGCACACAGG<br>TAAGCTT
Fas (XhoI/HindIII)	CTCGAGCAGAAGACAATGCTGCAGGATGTCCCCCCACTCTCTCTCT<br>CTCTCTCTCTCTCTCTCTCTCTCTCTCTCTCTCTCTCTCTCCATTCTGT<br>GTGTGTGTGTGTATTTGTATATTCTTAATTAGATAGATCCTCCAGGG<br>AGGAAAAGAAGTAGAAACAGAAGCTGAGCGAGCGATCAGTGGATA<br>AGCTT
Fas mutated RUNX1 (XhoI/HindIII)	CTCGAGCAGAAGACAATGCTGCAGGATGTCCCCCCACTCTCTCTCT<br>CTCTCTCTCTCTCTCTCTCTCTCTCTCTCTCTCTCTAGCTCGATCAGTAC<br>TGACGATCAGTCATAGCTAGCATGCCCTAGCCCTAGCGAGAGTTCA<br>CGAGGAAAAGAAGTAGAAACAGAAGCTGAGCGAGCGATCAGTGGA<br>TAAGCTT
Lipin1 (XhoI/HindIII)	CTCGAGTCAGATTTGGTGGGAAACTCATAGAGTAAACCATAGAGAG<br>TATGGATTTTTGTTGTTGCTGTGTGATCTTCTGTGTGTAGTGTGTGTG<br>TGTGTGTGTGTGTGTGTGTGTGTGTGTGTGTGTGTGTGTGTGAATAA<br>TGGTACATGCATTCCACAGCACGGAAGTAAGAGGACCACCAACCA<br>CTTGGAAGCTT
Lipin1 mutated RUNX1 (XhoI/HindIII)	CTCGAGTCAGATTTGGTGGGAAACTCATAGAGTAAACCATAGAGAG<br>TATGGATTTTTGTTACGAGTCGTCATGCATCGAGCTACTGAGCTGAC<br>GACTGACATGAAGCTACTGAAGCTAGCTACCCAGACCCCCGATAGA<br>CCCCCGGAATGCATTCCACAGCACGGAAGTAAGAGGACCACCAAC<br>CACTTGGAAGCTT
Lpl (XhoI/HindIII)	CTCGAGGGCAGAAGGACAGACAGAATTTCCTTACTCTTTGGTTTTG<br>GAACACCCCCCCCCCTCCACACACACACACACACACACACACACACA<br>CACACACACACACACACACACTTCCCACAAGGTACTAAGGTGCATA<br>TTTTACATATCAAAACAGACCTGGCCTCCAGGTTCTCCCAGCATCCC<br>TAAGCTT
Lpl mutated RUNX1 (XhoI/HindIII)	CTCGAGGGCAGAAGGACAGACAGAATTTCCTTACTCTTTGGTTTTG<br>GAACACCCCCCCCCAGTCGCTAGCGAGCTTAGAGGCTCGCTAGAGC

	CGCAATGCGAGGCTACTGACCGTCAGGAAAGGTACTAAGGTGCAT ATTTTACATATCAAAACAGACCTGGCCTCCAGGTTCTCCCAGCATCC CTAAGCTT
Pparγ (XhoI/HindIII)	CTCGAGCACTGAAGGCAGTAGAATTACAAATTCTAGTCTGGGCTTT ATAAAGAGACACTATCACAAACAAACAGACAAAGGAAGGAAATAA TGCCAGCTACAACCCAGGTGGGCTTTGACAACATCATGCTAAGCAA AGGAAACCAATCATACAAGACATTGTATTAAGCTT
Pparγ mutation (XhoI/HindIII)	CTCGAGCACTGAAGGCAGTAGAATTACAAATTCTGACTCAAATCCC GCGGGAGAGTGACGCAGTAGATGCGTCAGGTCATTCCTTCCCGCCG TAAAGCTACAACCCAGGTGGGCTTTGACAACATCATGCTAAGCAAA GGAAACCAATCATACAAGACATTGTATTAAGCTT
Scd1 (XhoI/HindIII)	CTCGAGCAGAAGAAAGAAAGGAACTATGTAGAGTAGGCTGCAAAG CCATTGATATCAGACAGACAGACAGACAGGCAGGCAGGCAGGCAG GCAGAAGAAAAGCGAGAAGAGGAAAAAAAGAAAAGAACTCACTA TGTAGACCAGGCTGCATTTGGCAAGCTT
Scd1 mutated RUNX1 (XhoI/HindIII)	CTCGAGCAGAAGAAAGAAAGGAACTATGTAGAGTAGGCTGCAAAA TTGCCAGCGCTGAGTGAGTGAGTGAGTGAATGAATGAAGGAATGA ATGAGGAGGGGATAGGAAGAGGAAAAAAAGAAAAGAACTCACTA TGTAGACCAGGCTGCATTTGGCAAGCTT
Srebf1 (XhoI/HindIII)	CTCGAGAACTCACTTAGCACATGGGCTTCCCTTTCTGTACATCCTAT ACTATTTTTCTATAAGGGCTCTTGACACCTCAAAACCAAAAACAAA CAAACAAACAAAACCCTGGCAGATGGCGAATCAGCTCGCACCAGA TGTGGACTCTAATGAGACCAACCCTCATCTAGGGAAGCTT
Srebf1 mutated RUNX1 (XhoI/HindIII)	CTCGAGAACTCACTTAGCACATGGGCTTCCCTTTCTGTACATCCTAT ACTATTTTTCTCGCCTTTAGAGGTCAGCAAGCCCCAACCCGGACCC ACCGAGCCGACGCGAACATGAGATGCCGAATCAGCTCGCACCAGA TGTGGACTCTAATGAGACCAACCCTCATCTAGGGAAGCTT
ZBTB7B (XhoI/HindIII) (RUNX1 control)	CTCGAGAGGGACCTGGACTCCGCCCCTCGTGCGCGGGGATCCCGGG TCAGCCCGGGTGAGGGCCAGGGCCCCCCTGGCGCCGCCCATTGTGG TCCAACAGGTTGAGCTGGTGTCCCGAGAAGCCACCTGCTGGAAAGG GAGGGAGGCCCCCTGCGGAGGGGGGTTGGGAGGGGAAAGCGAGTC CGAAAGCTT
ZBTB7B-mutated RUNX1 (XhoI/HindIII) (RUNX1 control mutated)	CTCGAGAGGGACCTGGACTCCGCCCCTCGTGCGCGGGGATCCCGGG TCAGCCCGGGTGAGGGCCAGGGCCCCCCTGGCGCCGCCCATCACAA CCCAACAGGTTGAGCTGGTGTCCCGAGAAGCCACCTGCTGGAAAGG GAGGGAGGCCCCCTGCGGAGGGGGGTTGGGAGGGGAAAGCGAGTC CGAAAGCTT
