# Supplementary material for: Erythropoietin regulates energy metabolism through EPO-EpoR-RUNX1 axis
Source: Nat Commun. 2024 Sep 16;15:8114. doi: 10.1038/s41467-024-52352-z (PMC11405798; doi:10.1038/s41467-024-52352-z)
Supplement: Supplementary file 3 — Reporting Summary [file 41467_2024_52352_MOESM3_ESM.pdf]

nature portfolio

Corresponding author(s): Constance Tom Noguchi

Last updated by author(s): 07/28/2024

Reporting Summary

Nature Portfolio wishes to improve the reproducibility of the work that we publish. This form provides structure for consistency and transparency in reporting. For further information on Nature Portfolio policies, see our Editorial Policies and the Editorial Policy Checklist.

Statistics

For all statistical analyses, confirm that the following items are present in the figure legend, table legend, main text, or Methods section.

n/a	Confirmed	
☐	☒	The exact sample size (*n*) for each experimental group/condition, given as a discrete number and unit of measurement
☐	☒	A statement on whether measurements were taken from distinct samples or whether the same sample was measured repeatedly
☐	☒	The statistical test(s) used AND whether they are one- or two-sided<br>*Only common tests should be described solely by name; describe more complex techniques in the Methods section.*
☐	☒	A description of all covariates tested
☐	☒	A description of any assumptions or corrections, such as tests of normality and adjustment for multiple comparisons
☐	☒	A full description of the statistical parameters including central tendency (e.g. means) or other basic estimates (e.g. regression coefficient) AND variation (e.g. standard deviation) or associated estimates of uncertainty (e.g. confidence intervals)
☐	☒	For null hypothesis testing, the test statistic (e.g. *F*, *t*, *r*) with confidence intervals, effect sizes, degrees of freedom and *P* value noted<br>*Give P values as exact values whenever suitable.*
☒	☐	For Bayesian analysis, information on the choice of priors and Markov chain Monte Carlo settings
☒	☐	For hierarchical and complex designs, identification of the appropriate level for tests and full reporting of outcomes
☒	☐	Estimates of effect sizes (e.g. Cohen's *d*, Pearson's *r*), indicating how they were calculated

Our web collection on statistics for biologists contains articles on many of the points above.

Software and code

Policy information about availability of computer code

Data collection	N/A
Data analysis	Immunofluorescence were taken by Zeiss 880 confocal microscopy, ZEN software were used to analysis the images.<br>The quantitative real-time RT-PCR were conducted using a 7900 Sequence Detector (PE Applied Biosystems, Foster City, CA, USA), analyzing with the Delta-Delta CT method.

For manuscripts utilizing custom algorithms or software that are central to the research but not yet described in published literature, software must be made available to editors and reviewers. We strongly encourage code deposition in a community repository (e.g. GitHub). See the Nature Portfolio guidelines for submitting code & software for further information.

Data

Policy information about availability of data

All manuscripts must include a data availability statement. This statement should provide the following information, where applicable:

- Accession codes, unique identifiers, or web links for publicly available datasets
- A description of any restrictions on data availability
- For clinical datasets or third party data, please ensure that the statement adheres to our policy

All the data supporting the findings of this study are available within the article and its supplementary information files, the original data was uploaded in the Figshare database. A reporting summary for this article is available as a Supplementary Information file. The source data underlying Figures and Supplementary

Figures are provided as a Source Data file.

Research involving human participants, their data, or biological material

Policy information about studies with human participants or human data. See also policy information about sex, gender (identity/presentation), and sexual orientation and race, ethnicity and racism.

Reporting on sex and gender	N/A
Reporting on race, ethnicity, or other socially relevant groupings	N/A
Population characteristics	N/A
Recruitment	N/A
Ethics oversight	N/A

Note that full information on the approval of the study protocol must also be provided in the manuscript.

Field-specific reporting

Please select the one below that is the best fit for your research. If you are not sure, read the appropriate sections before making your selection.

[x] Life sciences [ ] Behavioural & social sciences [ ] Ecological, evolutionary & environmental sciences

For a reference copy of the document with all sections, see nature.com/documents/nr-reporting-summary-flat.pdf

Life sciences study design

All studies must disclose on these points even when the disclosure is negative.

Sample size	All experiments were replicated at least three times independently. For all experiments, the investigators were blinded to group allocation during data collection and analysis.<br>Most of the samples were list in the experiment method,<br>For qRT-PCR, each group were use 5 mice.<br>For ELISA experiments, each group use 6 or 8 mice serum samples.<br>For immunohistology experiments, at least 3 mice tissue were processed for section and stained with antibodies. For each staining, at least 10 images were taken randomly, one image were shown in the figure.<br>For Western blot analysis, all of the experiments were repeated 3 times from protein preparation and 1 representative blot was shown in the figure.
Data exclusions	No data were excluded from the analyses.
Replication	All attempts at replication were successful. Each experiment was replicated at least three times independently, and one set of results is shown in the figure.
Randomization	All the mice were randomly separated into different groups. Littermates were used as control when available.
Blinding	The mice metabolism assay was conducted by an individual unaware of the mice genotype. Post-experiment, mice were categorized based on their ear tag numbers. IHC and IF imaging, carried out by an individual unaware of the genotype, was subsequently matched with slides bearing genotype indicators for result analysis.

Reporting for specific materials, systems and methods

We require information from authors about some types of materials, experimental systems and methods used in many studies. Here, indicate whether each material, system or method listed is relevant to your study. If you are not sure if a list item applies to your research, read the appropriate section before selecting a response.

Materials & experimental systems

n/a	Involved in the study
☐	☒ Antibodies
☐	☒ Eukaryotic cell lines
☒	☐ Palaeontology and archaeology
☐	☒ Animals and other organisms
☒	☐ Clinical data
☒	☐ Dual use research of concern
☒	☐ Plants

Methods

n/a	Involved in the study
☒	☐ ChIP-seq
☒	☐ Flow cytometry
☒	☐ MRI-based neuroimaging

Antibodies

Antibodies used

For IHC, IF and WB:
Goat Anti-Erythropoietin R Antibody, Novus Biologicals, Cat#AF1390;
Rabbit RFP Antibody Pre-adsorbed, ROCKLAND, Cat#:600-401-379;
Mouse RUNX1 (middle) Polyclonal Antibody,ThermoFisher,Cat#25315-1-AP;
Mouse Anti-UCP1 Antibody (536435), Novus Biologicals, Cat#MAB6158;
Rabbit Anti- K48-linkage Specific Polyubiquitin Antibody, Cell signaling, Cat#4289;
Rabbit Anti-K63-linkage Specific Polyubiquitin (D7A11) Rabbit mAb, Cell signaling, Cat#5621;
Rabbit Anti-FBXW7 Polyclonal Antibody, ThermoFisher, Cat#40-1500;
Rabbit DYKDDDDK Tag (D6W5B) Rabbit mAb (Binds to same epitope as Sigma's Anti-FLAG® M2 Antibody), Cell signaling, Cat#14793;
Mouse Myc-Tag (9B11) Mouse mAb, Cell signaling, Cat#2276;
Rabbit anti-CBF-beta (isoform 1) Antibody, FORTIS LIFE SCIENCES, Cat#A303-549A;
Rabbit anti-β-Actin Antibody, Cell signaling, Cat#4967;
Mouse anti-GAPDH Antibody (1D4); ThermoFisher, Cat#:MA1-16757;
Goat anti-Mouse IgG (H+L) Secondary Antibody,HRP ThermoFisher, Cat#31430;
Goat anti-Rabbit IgG (H+L) Secondary Antibody, HRP, ThermoFisher, Cat#31460;
Rabbit anti-Goat IgG (H+L) Secondary Antibody, HRP,ThermoFisher, Cat#31402;
Donkey anti-Goat IgG (H+L) Cross-Adsorbed Secondary Antibody, Alexa Fluor™ 488, Novus Biologicals, Cat#NB7356;
Goat anti-Mouse IgG (H+L) Cross-Adsorbed Secondary Antibody, Alexa Fluor™ 488, ThermoFIsher, Cat#A-11001;
Goat anti-Rabbit IgG (H+L) Cross-Adsorbed Secondary Antibody, Alexa Fluor™ 568, ThermoFIsher, Cat#A-11011;
Goat anti-Mouse IgG (H+L) Secondary Antibody [FITC], Novus Biologicals, Cat#NB7538;

Validation

For IHC, IF and WB:
Goat Anti-Erythropoietin R Antibody, Novus Biologicals, Cat#AF1390;Applications: WB, 1:1000; ICC/IF,1:100; Reactivity:M;
Rabbit RFP Antibody Pre-adsorbed, ROCKLAND, Cat#:600-401-379; Applications: WB:1:1000; IHC/IF: 1:200; Reactivity:RFP, mScarlet, rRFP, tdTomato;
Mouse RUNX1 (middle) Polyclonal Antibody,ThermoFisher,Cat#25315-1-AP;Applications:WB: 1:1000; IHC, IHC(P), ICC/IF, 1:100; IP,1:100; Reactivity:M, H, R;
Mouse Anti-UCP1 Antibody (536435), Novus Biologicals, Cat#MAB6158;Applications:Flow, ICC/IF, 1:200; Reactivity:M,H;
Rabbit Anti- K48-linkage Specific Polyubiquitin Antibody, Cell signaling, Cat#4289;Applications:WB, 1:1000;
, Reactivity:M,H, R, Mk, Vir, Mi, C, Dm, X, Z, B, Dg, Pg, Sc, Ce, Hr, Gp, Rab;
Rabbit Anti-K63-linkage Specific Polyubiquitin (D7A11) Rabbit mAb, Cell signaling, Cat#5621;Applications:WB, 1:1000; Reactivity:M,H, R, Mk, Vir, Mi, C, Dm, X, Z, B, Dg, Pg, Sc, Ce, Hr, Gp, Rab;
Rabbit Anti-FBXW7 Polyclonal Antibody, ThermoFisher, Cat#40-1500;Applications:WB, 1:1000; Reactivity:M,H;
Rabbit DYKDDDDK Tag (D6W5B) Rabbit mAb (Binds to same epitope as Sigma's Anti-FLAG® M2 Antibody), Cell signaling, Cat#14793;Applications:WB, 1:2000; IP, 1:200; IHC, Chip, Flow, IF, 1:200; Reactivity:M,H, R, Mk, Vir, Mi, C, Dm, X, Z, B, Dg, Pg, Sc, Ce, Hr, Gp, Rab;
Mouse Myc-Tag (9B11) Mouse mAb, Cell signaling, Cat#2276;Applications:WB, 1:2000; IP, IHC, Chip, Flow, IF, 1:100; Reactivity:M,H, R, Mk, Vir, Mi, C, Dm, X, Z, B, Dg, Pg, Sc, Ce, Hr, Gp, Rab;
Rabbit anti-CBF-beta (isoform 1) Antibody, FORTIS LIFE SCIENCES, Cat#A303-549A; Applications:WB, 1:1000; Reactivity:M,H;
Rabbit anti-β-Actin Antibody, Cell signaling, Cat#4967; Applications:WB, 1:5000; Reactivity:R, H, M, Hm,Mk, Mi, Dm, Z, B;
Mouse anti-GAPDH Antibody (1D4); ThermoFisher, Cat#:MA1-16757; Applications:WB, 1:3000; Reactivity: Avian, Bovine, Dog, Chicken, Fruit fly, Horse, Cat, Hamster, Human, Marsupial, Mouse, Non-human primate, Sheep, Pig, Rabbit, Rat, Zebrafish;
Goat anti-Mouse IgG (H+L) Secondary Antibody,HRP ThermoFisher, Cat#31430 Applications:WB, 1:3000; Reactivity:M;
Goat anti-Rabbit IgG (H+L) Secondary Antibody, HRP, ThermoFisher, Cat#31460;Applications:WB, 1:2500; Reactivity:R;
Rabbit anti-Goat IgG (H+L) Secondary Antibody, HRP,ThermoFisher, Cat#31402;Applications:WB,1:4000; IP, IHC, IHC(P), ICC/IF, 1:300; Reactivity:G;
Donkey anti-Goat IgG (H+L) Cross-Adsorbed Secondary Antibody, Alexa Fluor™ 488, Novus Biologicals, Cat#NB7356; Applications: IF/IHC, 1:300;
Goat anti-Mouse IgG (H+L) Cross-Adsorbed Secondary Antibody, Alexa Fluor™ 488, ThermoFIsher, Cat#A-11001; Applications: IF/IHC, 1:300;
Goat anti-Rabbit IgG (H+L) Cross-Adsorbed Secondary Antibody, Alexa Fluor™ 568, ThermoFIsher, Cat#A-11011; Applications: IF/IHC, 1:300;
Goat anti-Mouse IgG (H+L) Secondary Antibody [FITC], Novus Biologicals, Cat#NB7538;Applications:Flow, IHC, ICC/IF, 1:200;

Reactivity:M;

Eukaryotic cell lines

Policy information about cell lines and Sex and Gender in Research

Cell line source(s)	3T3-L1, ATCC, CL-173™;<br>293T/17 [HEK293T/17], ATCC, CRL-11268;
Authentication	All cell lines were authenticated by the company from which the line was purchased.
Mycoplasma contamination	All of the cell lines have been tested and were negative for mycoplasma contamination.
Commonly misidentified lines (See ICLAC register)	No commonly misidentified lines were used.

Animals and other research organisms

Policy information about studies involving animals; ARRIVE guidelines recommended for reporting animal research, and Sex and Gender in Research

Laboratory animals	Mice were maintained under a 12-hour light/dark cycle with free access to food and water. The animal room was kept at a constant temperature of 23°C with 40% humidity. All the male mice were used at the age of 6 to 8 weeks, this is not observed in female mice that are not ovariectomized due to the protective effect of estrogen to diet-induced obesity- detailed as follows: All mice was used in this study are C57BL/6J genetic backgorund. Mouse:C57BL/6J, Jackson Laboratories,Cat#000664; Tg6 mice, overexpressing PDGFbeta promoter-driven human erythropoietin transgene: Breeding of the resulting transgenic mouse line termed TgN(PDGFBEPO)321Zbz was performed by mating hemizygous males to wild-type females, thereby giving rise to heterozygous and wild-type littermates, the latter being used as controls; EpoR-tdTomato-Cre mice were generated by using CRISPR/Cas9 technology at Biocytogen; Mice with EpoR restricted to erythroid tissue (ΔEpoRE) contain the TgEpoRE transgene (mouse EpoR cDNA driven by GATA-1 hematopoietic regulatory domain) on a EpoR-/- background; EpoRAdiponecin-KO mice were generated by crossing Adiponectin-Cre mice (Strain #:028020 C57BL/6 background) with EpoRfloxp/floxp mice that were backcrossed onto a C57BL/6 background; EpoRaP2KO mice were generated by crossing aP2-Cre mice (Strain #:005069, C57BL/6 background) with EpoRfloxp/floxp mice. We only used the male mice for this study due to the female mice ovary produce estrogen has an protective effect on diet-induced obesity. This phenomena has been reported by another paper named "Sex difference in mouse metabolic response to erythropoietin. FASEB. J. 31, 2661-2673 (2017).", so we exclude the female mice from this study and only used the male mice.
Wild animals	The study did not involve wild animals.
Reporting on sex	Only males mice were used in the current study since estrogen provides protection against diet induced obesity in female mice and blunts the anti-obesity activity of erythropoietin.
Field-collected samples	The study did not involve samples collected from the field.
Ethics oversight	All breeding and experimental procedures were performed in accordance with the National Institutes of Health (NIH) Animal Research Advisory Committee Guidelines and with the approval of the ACUC at NIDDK-Bethesda, NIH.

Note that full information on the approval of the study protocol must also be provided in the manuscript.
